# Ethyl acetate extract of *Wedelia chinensis* inhibits tert-butyl hydroperoxide-induced damage in PC12 cells and D-galactose-induced neuronal cell loss in mice

**DOI:** 10.1186/1472-6882-14-491

**Published:** 2014-12-15

**Authors:** Wea-Lung Lin, Shao-Ming Wang, Ying-Jui Ho, Hsing-Chun Kuo, Yean-Jang Lee, Tsui-Hwa Tseng

**Affiliations:** Department of Pathology, Chung Shan Medical University Hospital, No. 110, Section 1, Chien-Kuo N. Road, Taichung, 402 Taiwan; School of Medicine, Chung Shan Medical University, No. 110, Section 1, Chien-Kuo N. Road, Taichung, Taiwan; School of Medical Applied Chemistry, Chung Shan Medical University, No. 110, Section 1, Chien-Kuo N. Road, Taichung, 402 Taiwan; School of Psychology, Chung Shan Medical University, No. 110, Section 1, Chien-Kuo N. Road, Taichung, 402 Taiwan; Department of Nursing, Chang Gung University of Science and Technology, Chia-Yi Campus No. 2, Chia-Pu Rd. West Sec. Putz, Chia-Yi, 613 Taiwan; Department of Chemistry, National Changhua University of Education, No. 1, Jin-De Road, Changhua, 500 Taiwan; Department of Medical Education, Chung Shan Medical University Hospital, No. 110, Section 1, Chien-Kuo N. Road, Taichung, 402 Taiwan

**Keywords:** Apoptosis, t-butylhydroperoxide, D-galactose, Luteolin, *Wedelia chinensis*, Wedelolactone

## Abstract

**Background:**

*Wedelia chinensis* is traditionally used as a hepatoprotective herb in Taiwan. The aim of this study was to evaluate the neuroprotective potential of *W. chinensis.*

**Methods:**

An ethyl acetate extract of *W. chinensis* (EAW) was prepared and analyzed by HPLC. The neuroprotective potential of EAW was assessed by tert-butylhydroperoxide (t-BHP)-induced damage in PC12 cells and D-galactose-induced damage in mouse cortex.

**Results:**

EAW exhibited potent radical scavenging property and highly contained luteolin and wedelolactone. EAW decreased t-BHP-induced reactive oxygen species (ROS) accumulation, cytotoxicity and apoptosis in PC12 cells. EAW and its major constituents blocked t-BHP-induced cytochrome C release and Bcl-2 family protein ratio change. EAW and its major constituents increased the endogenous antioxidant capacity evaluated by the binding activity assay of nuclear factor E2-related factor 2 (Nrf2) to antioxidant response element (ARE) and nuclear translocation of Nrf2 respectively in PC12 cells. Finally, EAW inhibited D-galactose-induced lipid peroxidation, apoptosis and neuron loss in the cerebral cortex of mice.

**Conclusion:**

These results demonstrate that *W. chinensis* has neuroprotective potential through blocking oxidative stress-induced damage and that luteolin and wedelolactone contribute to the protective action.

## Background

Oxidative tress occurs when the production of reactive oxygen species (ROS) or their products are in excess of antioxidant defense mechanism. The brain is especially vulnerable to oxidative stress compared to other organs because it exhibits lower antioxidant enzyme activity and contains an abundance of unsaturated fatty acids, which are the targets of lipid peroxidation. Thereafter oxidative stress contributes to the neurodegenerative disorders, and is one of the earliest pathological changes in Alzheimer’s disease [1]. Apart from quenching free radicals, antioxidants may combat the ROS generation and block ROS-mediated deleterious effects by gene regulation. Supplementations with exogenous antioxidants from diets as well as medicinal herbs catch attention on avoid unwanted neuropathophysiology.

Tertiary butyl hydroperoxide (t-BHP), a simple lipophilic alkyl hydroperoxide, has been reported to induce dose-dependent oxidative stress and damage in brain cells [2]. The alkoxyl and alkyl radicals derived from t-BHP are a species that produce cell damage [3, 4]. Furthermore, t-BHP-induces a cellular redox imbalance may induce cell apoptosis [5]. Therefore, t-BHP is a useful inducer for investigating the effectiveness of various neuroprotection products against oxidative stress. In addition, D-galactose at normal concentrations can be metabolized by D-galactokinase and galactose-1-phosphate uridyltransferase. However, high concentrations are converted into aldose and hydroperoxide by galactose oxidase, resulting in the generation of ROS in the brain [6, 7], which can affect bimolecules including protein, DNA and lipids and which may lead to consecutive functional disturbance and cell death. Thus, treatment of animals with D-galactose may serve as a useful model for studying the effect of neuroprotective agents.

*Wedelia chinensis,* a *Wedelia* genus plant belonging to the *Compositae*, has been traditionally used as a hepatoprotective tea material in Taiwan [8]. *W. chinensis* possesses multiple activities such as anti-microbial, anti-inflammatory, anti-cancer, and CNS-depressant activity [9–11]. Previously, Lin reported that *W. chinensis* contained four compounds capable of suppressing androgen activity: luteolin, apigenin, indole-3-carboxyaldehyde and wedelolactone [12]. Luteolin and apigenin, belonging to flavonoid structure, have been reported to reveal anti-oxidant, anti-inflammatory, and anti-cancer effects [13–15]. Wedelolactone, belonging to coumarin structure, has been demonstrated to exhibit a wide range of biological effects including anti-inflammation, immunomodulatory, anti-myotoxic, anti-oxidant, anti-phlogistic, anti-haemorrhagic, anti-hepatotoxic and anti-cancer activity [16–20]. Since the neuroprotection potential of *W. chinensis* is not well understood, in the present study we used two models, *in vitro* and *in vivo*, to evaluate its neuroprotective potential.

## Methods

### Reagents and materials

Dulbecco’s modified Eagle’s medium (DMEM), fetal bovine serum, and penicillin/streptomycin were purchased from GIBCO Ltd. (Grand Island, NY, USA). Antibodies, such as anti-Bcl-2, -Bcl-X_L,_ -Bax, -Nrf2 and -β-actin antibodies were obtained from Santa Cruz Biotechnology (Santa Cruz, CA). The herb, *W. chinensis*, was purchased from a reputable folk medicine store in Taiwan and identified by associate professor Ko-Kaung Liao, who majors in Biology and is familiar with medicinal plants. Wedelolactone, 7-methoxy-5, 11, 12-trihydroxy-coumestan, was synthesized from commercially available phloroglucinol [21]. All chemicals were analytical grade purchased from Sigma-Aldrich (USA) and Merck (Germany).

### Preparation of the crude extract

The air-dried whole plant of *W. chinensis* was shredded in a blender and extracted with 5 volumes of 95% ethanol at room temperature for 2 days. The mixture was filtered through filter paper (5 μm pore size), and the filtrate was dried using rotary evaporation under vacuum at 40°C. The percentage yield was 9.3% (w/w). The crude ethanolic extract was suspended in distilled water and partitioned successively with n-hexane and ethyl acetate, respectively to obtained semi-crudes. All dried extracts, including ethanol extract, n-hexane extract (HEW), and ethyl acetate extract (EAW) were stored at -20°C prior to use in the following studies.

### DPPH radical scavenging assay

The 2,2-diphenyl-1-picrylhydrazyl (DPPH) method measures the reaction of the antioxidants with the stable DPPH radical in a methanol solution. Briefly, a 60 μM DPPH radical solution was freshly made in methanol. Various concentrations of the sample extracts were reacted with the DPPH radical solution (3 mL) for 45 min at room temperature, and the absorbance was measured at 517 nm. The affinity of the test material to quench the DPPH free radical was evaluated according to the equation scavenging % = (absorbance of control group–absorbance of the extract added group)/absorbance of control group × 100%.

### HPLC analysis

EAW was analyzed using a Hitachi L7100 HPLC system with a 5-μm ODS-Hypersil column (250 × 4.6 mm). The mobile phase was generated from solvent A [acetonitrile: H_2_O: acetic acid (90:10:3)] and solvent B [acetonitrile: H_2_O: acetic acid (10:90:3)] using the following gradient program: 0–3 min 100% solvent B, 3–8 min 85% solvent B, 8–15 min 80% solvent B, 15–40 min 60% solvent B. The detection wavelength was 360 nm, and the flow rate 0.8 mL/min. Quantization was carried out by the external standard method on the basis of the area at 360 nm using calibration curve of wedelolactone and luteolin.

### Cell culture

Adrenal pheochromocytoma (PC12) cells were maintained in DMEM medium containing 5% fetal bovine serum, 10% horse serum, and 100 U/mL penicillin and streptomycin in a 5% CO_2_ incubator at 37°C.

### Preparation of nuclear protein and analysis of antioxidant-response element (ARE) binding activity of nuclear factor E2-related factor 2(Nrf2)

PC12 cells (1 × 10^6^ cells/ml) were exposed to the indicated reagent or vehicle (0.1% DMSO) for 6 h. Nuclear extracts were harvested using NE-PER nuclear extraction reagent (Thermo Fisher Scientific, Rockford, IL, USA) according to the manufacture’s instructions. Nuclear protein concentrations were determined using the Bio-Rad protein assay reagent. The amount of Nrf2 available in the nucleus to bind AREs was determined using a TransAM Nrf2 Transcription Factor ELISA Kit (Active Motif Inc., Carlsbad, CA, USA) according to the manufacturer’s instructions. Briefly, nuclear extracts (2.5 μg) were added to wells that contained the immobilized consensus ARE oligonucleotide. A primary anti-Nrf2 antibody was added to each well, followed by a horseradish peroxidase-conjugated secondary antibody. The signal was detected at 450 nm, and Nrf/ARE binding activity is reported as A_450_.

### Intracellular ROS analysis

PC12 cells were pretreated with EAW for 24 h and then treated with or without 100 μM of t-BHP for 3 h. After the indicated treatments, PC12 cells were incubated with the fluorescent probe 2′,7′-dichlorofouorescein diacetate (DCFH-DA; 10 μM) for 30 min. DCFH-DA becomes highly fluorescent 2′,7′-dichlorofouorescein (DCF) upon oxidation by ROS, and then washed twice with PBS. Finally the fluorescence intensity of DCF was measured in a microplate reader with an excitation wavelength 485 nm and an emission wavelength of 535 nm.

### Determination of cytotoxicity by LDH release assays

Cells were seeded in 24-well plates at a density of 2 × 10^5^ cells/ml. PC12 cells were pretreated with EAW (0, 10, 25 and 50 μg/mL) for 24 h and then treated with or without 100 μM of t-BHP for 3 h. The cytotoxicity was quantitatively assessed by measuring the activity of the lactate dehydrogenase (LDH) released from the damaged cells into the culture medium. At the end of the treatment, the media was used for the LDH activity assay. The enzyme was determined by using an assay kit according to the manufacturer’s protocol. The absorbance of the samples was read at 490 nm using a microplate reader. The LDH release was proportional to the number of damaged PC12 cells.

### Sub-G_0_/G_1_ hypodiploid FACS analysis

After treatment, the cells were fixed with ice-cold 70% ethanol overnight. The cells were then centrifuged at 1500 g for 10 min and resuspended in PBS containing 0.5% Triton X-100, 0.1 mg/mL RNase, and 40 μg/mL propidium iodide (PI) at room temperature for 30 min. Cellular DNA content (10,000 cells per analysis) was determined by flow cytometric analysis of PI-labeled cells using a FACSCalibur flow cytometer (Becton Dickinson, San Jose, CA) equipped with a single argon-ion laser (488 nm). Cell cycle was analyzed using Modfit software (Verity Software House, Topsham, ME) and those cells in apoptosis were indicated by the substantially lower DNA content compared to the G_0_/G_1_ peak in the DNA histogram.

### Western blotting analysis

The cells were washed in PBS with 1 mM zinc ion and lysed in radioimmunoprecipitation assay (RIPA) buffer (50 mM Tris-buffer, 5 mM EDTA, 150 mM NaCl, 1% NP 40, 0.5% deoxycholic acid, 1 mM sodium orthovanadate, 81 μg/mL aprotinin, 170 μg/mL leupeptin, and 100 μg/mL PMSF; pH 7.5). The mixtures were mixed for 30 min at 4°C and centrifuged (10,000 g) for 10 min. The supernatants were collected as whole-cell extracts. The protein content was determined with the Bio-Rad protein assay reagent using bovine serum albumin as a standard. Protein samples (50 to 100 μg protein) were boiled, separated on an SDS polyacrylamide gel, electrophoretically transferred to nitrocellulose membranes (Amersham, Arlington Heights, IL) and blotted with the indicated primary antibodies. The proteins were visualized with horseradish peroxidase (HRP)-conjugated secondary antibodies (Zymed Laboratory, Inc., San Francisco, CA) followed by chemiluminescence (ECL-Plus; Santa Cruz Biotechnology). The relative photographic density was quantified using densitometry.

### Assessment of cytochrome C release

Cells were harvested and washed with ice-cold PBS at the end of treatment to prepare the cytosolic fraction. The cell pellet was resuspended in 500 μL buffer A (20 mM HEPES-KOH, pH 7.5, 10 mM KCl, 1.5 mM MgCl_2_, 1 mM sodium EDTA, 1 mM leupeptin and 1 μg/mL chymostatin) and homogenized using a Pyrex glass homogenizer and a type B pestle (40 strokes). The homogenate was centrifuged at 100,000 g (4°C for 1 h) to generate the cytosolic fraction. A Bio-Rad protein assay kit determined protein concentrations, and 25 μg of each fraction was separated using 15% SDS-PAGE. The proteins were transferred to an NC membrane, and then reacted sequentially with a primary antibody (anti-cytochrome C and anti-β-actin as internal controls) and a secondary peroxidase-conjugated antibody. Protein bands were revealed using enhanced chemiluminescence (ECL commercial kit).

### Animals and treatments

Five-week-old male ICR mice were purchased from GlycoNex Inc. (Taiwan) and kept in groups of three to four per cage in an animal room at a temperature of 22 ± 2°C, 12 h light–dark cycle, and relative humidity 50-70%. The mice were provided with rodent chow and water *ad libitum*. All experiments were approved by the Animal Care Committee of Chung Shan Medical University (IACUC approval No. 896). After adaptation for a week, the mice were randomly divided into 4 groups (n = 6). Group 1 served as the vehicle control and received daily subcutaneous (s.c.) injections of 0.1 mL of distilled water and three intraperitoneal (i.p.) injections/week of 1% DMSO in distilled water for 9 weeks. Group 2 received daily s.c. injections of 50 mg/kg of D-galactose in distilled water and three i.p. injections/week of 0.1 mL of 1% DMSO in distilled water for 9 weeks. Groups 3 and group 4 received the same s.c. injections of D-galactose plus three i.p. injections/week of 0.1 mL of 1% DMSO containing, respectively, 10 mg/kg or 25 mg/kg of EAW.

### Preparation of brain samples and measurement of lipid peroxidation

Mice were euthanized and the brain removed and dissected on an ice-bath plate. The right cerebral cortex was fixed in 10% paraformaldehyde for histological studies and the left cerebral cortex was homogenized in 10 volumes (v/w) of ice-cold saline containing a protease inhibitor cocktail (Sigma-Aldrich, MO, USA). The resultant homogenate was centrifuged and the supernatant was removed for lipid peroxidation determination. The amount of malondialdehyde (MDA) was measured by reaction with thiobarbituric acid and the absorbance read at 532 nm on a spectrophotometer.

### TUNEL staining and histopathological examination

The cerebral cortex samples were dehydrated, embedded in paraffin blocks after post-fixation, then sliced and the sections stained with terminal deoxynucleotidyl transferase dUTP nick end labeling (TUNEL). The TUNEL positive cells were visualized using the peroxidase-DAB reaction and counterstained with hematoxylin. Detailed procedures followed the manufacturer’s protocol for the in situ Apoptosis Detection Kit (In Situ Cell Death Detection Kit; Roche Diagnostics, Mannheim, Germany) according to the manufacturer’s instructions. The digital images were captured using a digital camera (Canon A640, Tokyo, Japan). Quantification of apoptotic cells was by measuring three randomly selected microscopic fields (100× magnification) for each slide. The percentage of TUNEL-positive cell (%) = 100 × (TUNEL-positive cells/total cells). In addition, the sections were stained with hematoxylin-eosin for light microscopic analysis. Large nuclei and dark staining neurons in the cerebral cortex were counted in five random fields per slide at 400× magnification and the density of neurons expressed as no./mm^2^.

### Statistical analysis

Statistical significance was determined using one-way analysis of variance (ANOVA) followed by Dunnett’s or Tukey’s post hoc test. *P* values less than 0.05 were considered statistically significant.

## Results and discussion

### Free radical scavenging activity and main constituent of EAW

**Figure 1:**
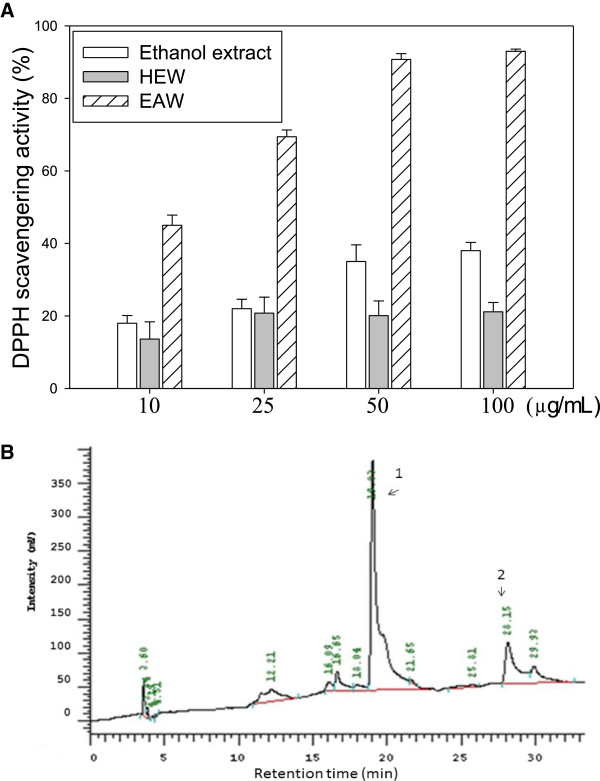
**DPPH quenching effect and HPLC analysis of** ***Wedelia chinesis*** **extract. (A)** The indicated concentrations of the ethanol extract, hexane extract (HEW), and ethyl acetate extract (EAW) of *Wedelia chinensis* were evaluated for DPPH quenching capacity as described in the text. % of DPPH scavengering activity = (absorbance of control group – absorbance of the extract added group)/absorbance of control group × 100%. Means ± SD (n = 3). **(B)** HPLC analysis of EAW*.* For the conditions, see the Materials and Methods. Peak 1 is wedelolactone, and peak 2 is luteolin.

Bleaching of DPPH was performed to determine the free radical scavenging capacity of the crude extracts. In our preliminary study, ethanol extract of *W. chinensis* exhibited free radical scavenging activity, and after solvent partition, it showed EAW increased the potency of free radical scavenging activity, with 10 μg/mL of EAW quenching ~50% of free radicals (Figure 1A). Then HPLC analysis examined the composition of EAW as compared with the authentic samples. This demonstrated that wedelolactone and luteolin were the main constituents (retention time 19.02 min and 28.15 min) (Figure 1B). The content of wedelolatone in EAW was 12.8% and luteolin was 11.4% respectively.

### EAW activates nuclear Nrf2-ARE binding activity

**Figure 2:**
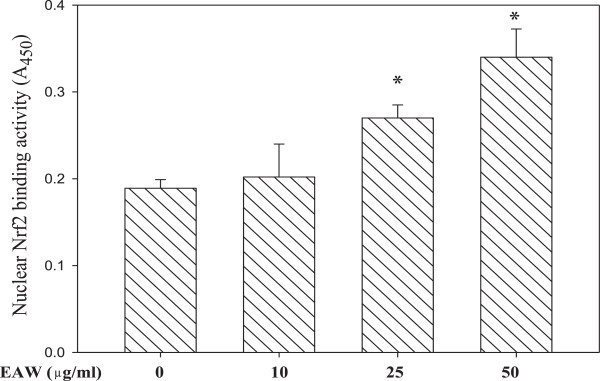
**Effects of EAW on Nrf2 activation.** After pretreatment with EAW, PC12 cells were harvested and nuclear extracts were prepared and analyzed using the TransAM Nrf2 kit, as described in the Materials and Methods. Data represent the means ± SD (n = 3). *P < 0.05, versus solvent control (0.1% DMSO) treated alone.

Figure 1 shows that EAW exhibited radical scavenging activity. In addition to direct scavenging radicals, whether noncytotoxic concentration of EAW has antioxidant enzyme induction activity in PC12 cells was investigated. The antioxidant response element (ARE) is a cis-acting enhancer sequence found in the promoter region of many genes encoding antioxidant enzymes/proteins. Nrf2 is a major ARE-binding transcription factor and induces the expression of antioxidant enzymes. Thereafter, whether EAW can enhance binding activity of nuclear Nrf2 to the ARE was evaluated. It was found that treatment with noncytotoxic concentration of EAW (25 and 50 μg/mL) significantly activated Nrf2–binding activity (Figure 2).

### Effects of EAW on t-BHP-induced intracellular ROS, cytotoxicity and apoptosis

**Figure 3:**
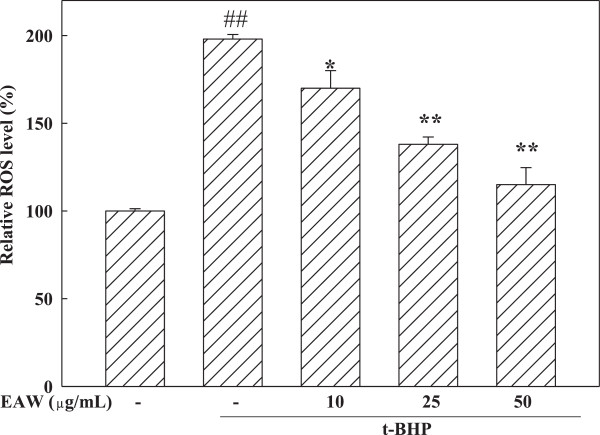
**Effects of EAW on t-BHP-induced intracellular ROS level.** After treatment, the intracellular ROS were determined using labeling with DCFH-DA as described in the text. ## *P* < 0.01, compared to the solvent control (0.1% DMSO). *P < 0.05 and **P < 0.01, compared to t-BHP treatment alone.

**Figure 4:**
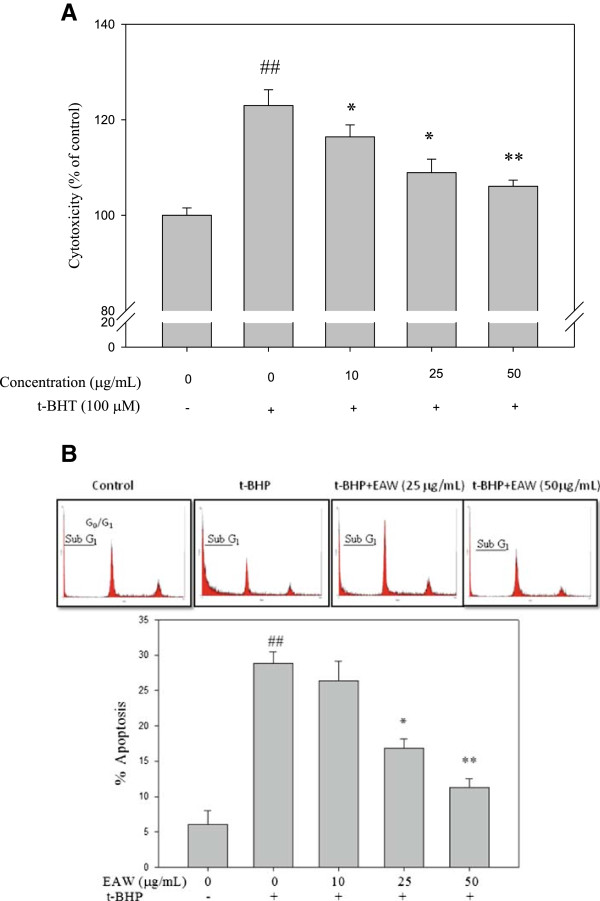
**Effects of EAW on t-BHP-induced cytotoxicity and apoptosis in PC12 cells.** PC12 cells were pretreated with EAW (0, 10, 25 and 50 μg/mL) for 24 h and then treated with or without 100 μM of t-BHP for 3 h. **(A)** The cytotoxicity was determined by the LDH assay kit. **(B)** The DNA content was analyzed by the flow cytometry as described in the text (upper panel). The percentage of apoptotic cells (sub-G_1_ phase) is expressed as mean ± SD (n = 3). ## *P* < 0.01, compared to the solvent control (0.1% DMSO). *P < 0.05 and **P < 0.01, compared to t-BHP treatment alone.

The intracellular level of ROS with or without EAW treatment prior to t-BHP exposure was evaluated. The treatment of PC12 cells with 100 μM t-BHP for 3 h increased DCF fluorescence. It implicated t-BHP induced intracellular ROS. In our preliminary study, pretreatment of EAW for 6 h didn’t exhibited apparent reduction effect on t-BHP-induced intracellular ROS (data not shown). However, pretreatment of EAW for 24 h showed reducing effect significantly (Figure 3). Thereafter, pretreatment of EAW for 24 h was used in the following cell culture study. LDH is a stable cytoplasmic enzyme that is present in all cells, including neurons. LDH is rapidly released into the cell culture medium when the cell plasma membrane is damaged. Therefore, LDH is a reliable biochemical index for neuronal cytotoxicity. Treatment with t-BHP for 3 h increased LDH release into the medium to 123.0 ± 3.3% of control (Figure 4A). Pretreatment with different EAW concentrations prior to t-BHP exposure significantly inhibited LDH leakage in the PC12 cell system compared to t-BHP treated alone group (Figure 4A). The flow cytometry analysis was used to evaluate the t-BHP-induced apoptosis of PC12 cells (Figure 4B). It showed t-BHP-induced 23.21% sub-G_0_/G_1_ phase, which indicated DNA degradation and the cells undergoing apoptosis. With the pretreatment of 25 and 50 μg/mL EAW significantly declined to 16.83% and 11.30% respectively compared to t-BHP treated alone group (Figure 4B).

### Effects of EAW and its major constituents on t-BHP-induced changes in cytochrome C and the Bcl-2 family

**Figure 5:**
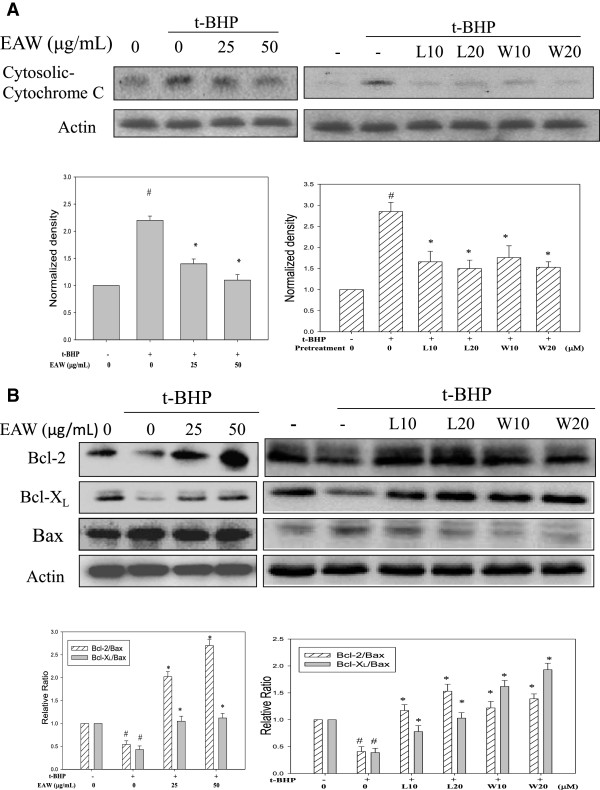
**Effects of EAW, luteolin and wedelolactone on t-BHP-induced cytochrome C release and changes in Bcl-2 family proteins.** PC12 cells were pretreated with EAW (25 and 50 μg/mL) or luteolin (L10, 10 μM; L20, 20 μM) or wedelolactone (W10, 10 μM; W20, 20 μM) for 24 h, then 100 μM of t-BHP was added for 3 h. **(A)** Cells were harvested after incubation, and the cytosolic protein extracts were prepared as described in the text and subjected to Western immunoblotting analysis against anti- cytochrome C. The average densitometric value of cytochrome C is shown as the mean ± SD (n = 3). # *P* < 0.05, compared to the solvent control (0.1% DMSO). *P < 0.05, compared to t-BHP treatment alone. **(B)** After treatment, the total cell lysates were prepared and subjected to Western immunoblotting analysis against against anti-Bcl-2, -Bcl-xL, -Bax and anti-actin. Actin was used as the loading control. The ratio of Bcl-2/Bax and Bcl-xL/Bax are shown as the mean ± SD (n = 3). # *P* < 0.05, compared to the solvent control (0.1% DMSO). *P < 0.05, compared to t-BHP treatment alone.

Oxidative stress initiates mitochondrial apoptotic signals [22, 23]. The loss of mitochondrial cytochrome C and its release into the cytoplasm are essential events in the initiation of mitochondrial caspase signaling. It was found that t-BHP treatment increased cytosolic cytochrome C, which was decreased by pretreatment with EAW or luteolin or wedelolactone (Figure 5A). The Bcl-2 family members have been found to play important roles in regulating mitochondrial-mediated apoptosis. High ratio of Bcl-2/Bax and Bcl-X_L_/Bax were observed to promote cell survival by preserving the integrity of the external mitochondrial membrane, which prevents the released of cytochrome C from the mitochondria, inducing cell death. It has been reported that overexpression of Bcl-2 in neurons was found to protect from neuronal loss in mouse model of cerebral ischemia [24]. In addition, Bax inhibition prevented neuronal death in a mouse model of Parkinson’s disease [25]. The ratio of Bcl-2/Bax and Bcl-X_L_/Bax are important in determining sensitivity to apoptotic stimuli. Therefore, we determined the effect of EAW, luteolin and wedelolactone on t-BHP–induced changes in these proteins. The treatment of PC12 cells with 100 μM t-BHP for 3 h decreased the ratio of Bcl-2/Bax and Bcl-X_L_/Bax significantly, which were reversed by the pretreatment of EAW or luteolin or wedelolactone (Figure 5B). However, PC12 cells cannot totally represent the characters of primary cultured neurons, so, the further work needs to focus directly on primary neurons.

### Effects of luteolin and wedelolactone on nuclear translocation of Nrf2 and γ-GCS expression

**Figure 6:**
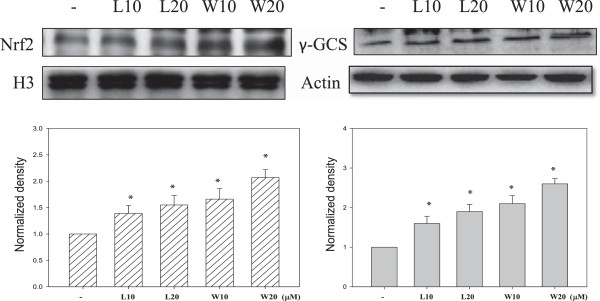
**Effects of luteolin and wedelolactone on Nrf2 nuclear translocation and γ-GCS expression.** The cells were treated with luteolin or wedelolactone for 24 h prior, then the cells were collected for protein extraction. The nuclear protein extract and total protein extract were applied to western blotting analysis against anti-Nrf2 and anti-γ-GCS respectively. The average densitometric value is shown as the mean ± SD (n = 3). *P < 0.05, compared to the solvent control (0.1% DMSO).

It has been demonstrated that some antioxidants are exerting their antioxidative effect by activating Nrf2, which is a master regulator of the antioxidant response [26]. Activation of Nrf2 requires its translocation into the nucleus. It showed luteolin and wedelolactone increased the nuclear translocation of Nrf2, which implicated activation of Nrf2 (Figure 6). In addition, luteolin and wedelolactone increased the protein expression of γ-glutamyl-cysteine synthetase (γ-GCS), a product of Nrf2 target gene (Figure 6). Luteolin and wedelolactone containing phenolic structure have been reported to exhibit free radical quenching properties [27, 28]. Therefore, luteolin as well as wedelolactone contributed to the neuroprotective potential of EAW by activating Nrf-2 and quenching ROS. In addition to oxidative stress, degenerative lesions are associated with inflammatory reaction [29]. Since luteolin and wedelolactone also have anti-inflammatory potential [13, 16], this may also contribute to the neuroprotective potential of *W. chinensis.*

### Effect of EAW on MDA levels and neuronal cell density in the cerebral cortex of D-galactose-treated mice

**Figure 7:**
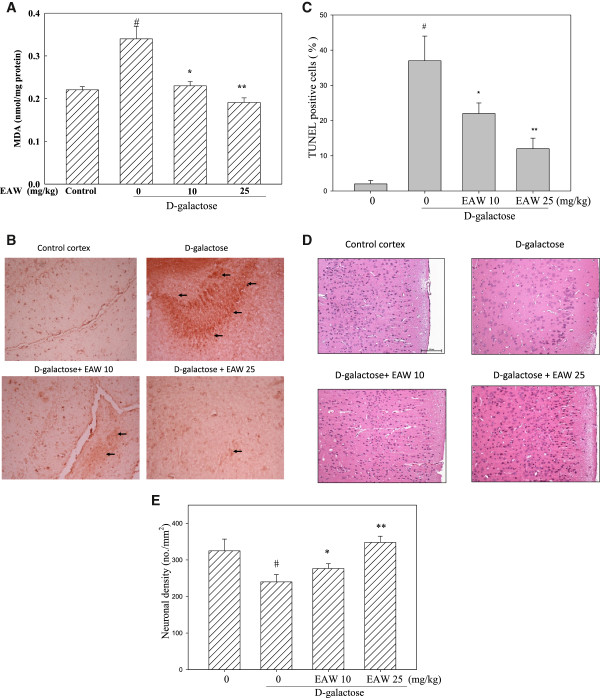
**Effects of EAW on lipid peroxidation, TUNEL assay, and neuronal density in the cortext of D-galactose-treated mice. (A)** MDA levels were analyzed as described in the text. **(B)** Representative of TUNEL stained brain section. Arrow indicates TUNEL positive cells. **(C)** TUNEL positive cells were quantified as described in the text. Data are expressed as mean ± SD (n = 6). **(D)** Representative hematoxylin-eosin stained sections of the cortex in each group (200×). Scale bar: 100 μm. **(E)** Neuronal density calculated from the number of neurons in a 0.218 mm^2^ field (400x) and expressed as the mean ± SD for the number of mice indicated in the text. # *P <* 0.05, compared to the control group; *P < 0.05 and **P < 0.01, compared to the D-galactose-treated group.

Oxidative stress is proposed to be major contributor of the aging process and is linked to neurodegenerative disease such as Alzheimer’s disease [30, 31]. Neurodegenerative diseases are characterized by progressive neuronal loss that leads to functional declines. There has been a growing interest in preventing various age-related pathologic conditions in brain. One of strategies to slow or delay neuronal damage is reducing brain oxidative stress. MDA is a metabolic product of lipid peroxidation, which is generated under oxidative stress. It has been reported that high concentration of D-galactose may induce oxidative stress in brain [7]. Figure 7A shows that D-galactose treatment caused an increase in MDA levels in the cortex compared to the control group (*P* < 0.05) and that this increase was reduced by co-administration of 10 or 25 mg/kg of EAW. Since oxidative stress might induce cell apoptosis we investigated the effect of D-galactose on cell apoptosis in the cortex by TUNEL assay. This showed TUNEL-positive cells were significantly increased in the D-galactose treated group compared to the vehicle control group (*P* < 0.05), and that they were reduced by co-administration of EAW (*P* < 0.05; Figure 7B and C). In addition, Figure 7D shows representative photomicrographs of hematoxylin-eosin staining in the cortex of control mice and mice treated with D-galactose with or without co-administration of EAW. Neuron cells (large nucleus and dark staining) in the sections are stained by hematoxylin-eosin. As shown in Figure 7E, cell counts revealed that mice treated with D-galactose for 9 weeks showed neuronal loss in the cortex compared to the vehicle control group (*P* < 0.05) and that this D-galactose-induced neuronal cell loss was inhibited by co-administration of EAW significantly (*P* < 0.05). One might be interested in knowing the dosage of EAW when applying in the human. By using the body surface area normalization method [32], the effective dose, 10 and 25 mg/kg, of EAW in preventing neuronal loss in the cortex in mice can be translated to the human equivalent dose, 0.81 and 2.03 mg/kg. However, further study is needed to evaluate the effective amount of *W. chinensis* when used in human for improving the health or for a therapeutic purpose.

## Conclusion

EAW suppressed t-BHP-induced cytotoxicity and apoptosis by decreasing intracellular ROS through scavenging radicals and activating endogenous antioxidant capacity in PC12 cells. *In vivo* experiment, EAW reduced D-galactose-induced lipid peroxidation and neuron apoptosis in the cerebral cortex of mice. Further, in addition to luteolin, we found wedelolactone, induced the nuclear translocation of Nrf2 and the expression of γ-GCS. These results demonstrate that *W. chinensis* possesses neuroprotective potential by blocking oxidative stress-induced cell damage and that luteolin and wedelolactone contribute to the action. Research on other mechanisms by which EAW exerts its neuroprotective effect is currently in progress.

## Abbreviations

ARE: Antioxidant response element
t-BHP: Tert-butylhydroperoxid
DPPH: 2,2-diphenyl-1-picrylhydrazyl
γ-GCS: γ-glutamyl-cysteine synthetase
LDH: Lactate dehydrogenase
Nrf2: Nuclear factor E2-related factor 2
ROS: Reactive oxygen species
TUNEL: Terminal deoxynucleotidyl transferase dUTP nick end labeling.
